# Imported Malaria and Congenital Acquisition in Infant, Portugal, 2024

**DOI:** 10.3201/eid3109.250536

**Published:** 2025-09

**Authors:** Inês Lopes, Joana Dias, Edvaldo Das Neves, Maria Morais, Ana Santos-Reis, Ana M. Garcia, Luis Varandas, Dinora Lopes

**Affiliations:** Global Health and Tropical Medicine, Associate Laboratory in Translation and Innovation Towards Global Health, Instituto de Higiene e Medicina Tropical, Universidade NOVA de Lisboa, Lisbon, Portugal (I. Lopes, E. Das Neves, A. Santos-Reis, L. Varandas, D. Lopes); Unidade Local de Saúde São José, Lisbon (J. Dias); Hospital of Vila Franca de Xira, Vila Franca de Xira, Portugal (M. Morais); Unidade Local de Saúde São José EPE Centro de Investigação, Lisbon (A.M. Garcia, L. Varandas)

**Keywords:** malaria, Plasmodium falciparum, parasites, congenital transmission, nonendemic area, subpatent parasitemia, Portugal

## Abstract

*Plasmodium falciparum* infection was diagnosed in a 3-month-old baby in Portugal by optical microscopy. The mother had had malaria in Angola 13 months earlier, before she emigrated to Portugal. She remained asymptomatic throughout and after pregnancy. We confirmed the diagnosis of an imported malaria case and congenital transmission using molecular techniques.

Imported malaria cases in Europe from sub-Saharan Africa countries can manifest with very low parasite densities and asymptomatic infections. Those infections can pose a public health threat, given the potential for onward transmission in areas with competent vectors and suitable conditions (*1*–*3*). 

Congenital malaria in infants is rare because the placenta acts as an effective barrier preventing the transfer of malaria parasites from maternal to fetal circulatory system; transmission during labor is the most likely mechanism (*4*). Ultrasensitive molecular diagnostic tools detect infections in settings with low parasite density (*5*,*6*). Congenital malaria is not universally defined. Some authors define it as the presence of asexual forms of malaria parasites in an infant’s cord blood or peripheral blood during the first week of life, regardless of clinical symptoms (*4*,*7*). Others require the presence of parasites in the newborn’s peripheral blood on the first day of life for diagnosis (*4*,*7*). However, a timely diagnosis can be missed if a patient has no suggestive symptoms or clinical or travel history that prompt an early assessment. We report *Plasmodium falciparum* infection in a 3-month-old baby in Portugal.

## The Study

A 3-month-old female infant was brought to a pediatric emergency department of Vila Franca de Xira Hospital (Vila Franca Xira, Portugal) with a 2-day history of fever and splenomegaly; maximum axillary temperature was 38.6°C. Initial laboratory tests revealed a hemoglobin level of 10.9 g/dL (reference threshold is 11.0 g/L for children 6–59 months of age), a leukocyte count of 10,500/mm^3^ (2,040/mm^3^ neutrophils and 5,970/mm^3^ lymphocytes) (reference ranges 7,300–16,600/mm^3^ for leukocytes, 1,500–6,900/mm^3^ for neutrophils, and 3,400–9,400/mm^3^ for lymphocytes), platelet count of 66,000/mm^3^ (reference range 180,000–440,000/mm^3^), and C-reactive protein level of 53.4 mg/L (reference range <10 mg/L). A peripheral blood smear revealed *P*. *falciparum* trophozoites; parasite count was estimated at 4.6%. Further investigation of family history revealed that the infant was born via cesarean delivery; infant and mother were discharged from hospital without any concerns. The infant had no history of traveling abroad, but her mother had moved to Portugal from Angola 13 months earlier. The mother had received artemether/lumefantrine treatment for *P. falciparum* infection in Angola 1 week before relocating to Portugal. 

Because our findings were consistent with a suspected case of congenital malaria, we obtained samples from the mother and infant for molecular testing. We collected samples from the infant, with written consent from her mother, from the neonatal Guthrie card with blood collected via heel prick at the fourth day of life, during initial hospital admission, and at a follow-up appointment, at which we also took a sample from the mother. Maternal thick and thin blood smears did not reveal any malarial parasites; a rapid diagnostic test for malaria returned negative results.

**Figure 1:**
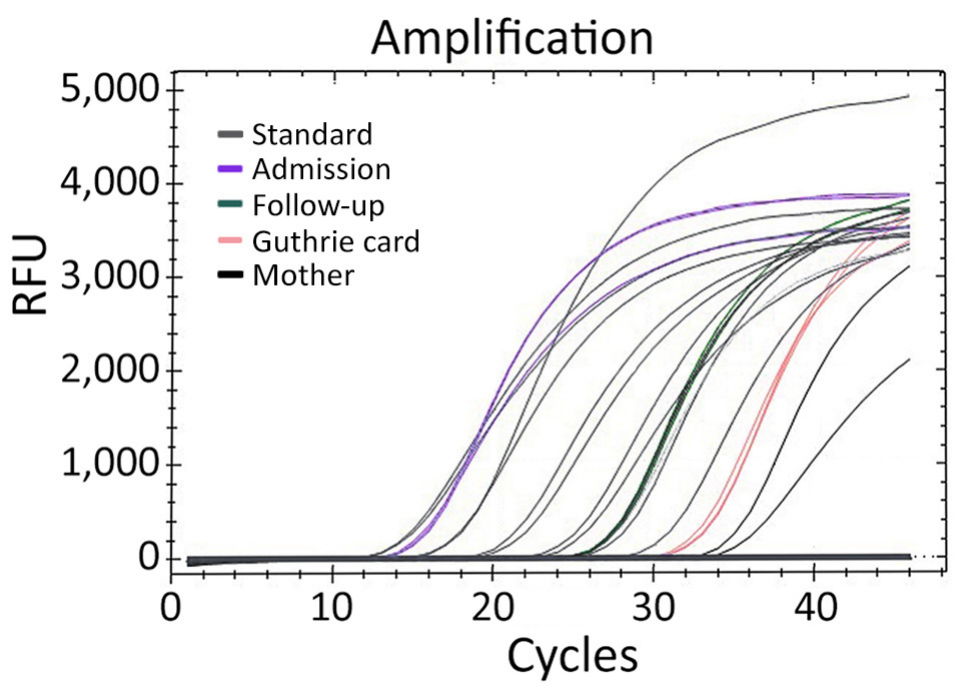
Quantitative PCR amplification of samples from infant and mother in study of congenital malaria, Portugal. We obtained results from arithmetic average values of triplicate results. Additional curves represent standard results obtained by serial dilution to determine parasite quantification. Results were 0.07 parasites/µL from infant’s Guthrie card sample 4 days after birth; 1,178.13 parasites/µL from sample taken at hospital admission; 21.99 parasites/µL from sample taken at follow-up appointment; 0.03 parasites/µL from sample taken from the mother at follow-up appointment. RFU, relative fluorescence unit.

**Figure 2:**
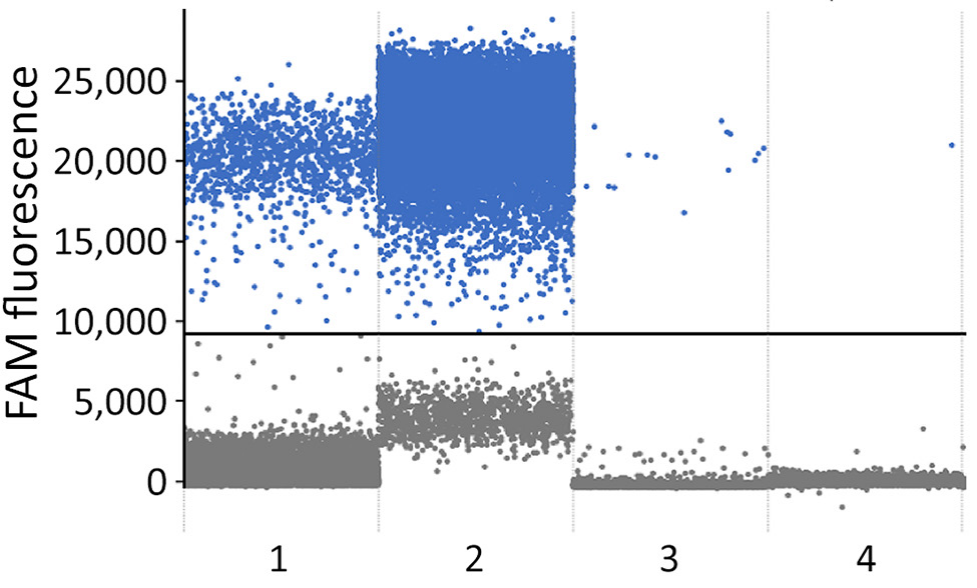
Digital PCR results from samples from infant and mother in study of congenital malaria, Portugal. We obtained results from arithmetic average values (parasite/µL) of triplicate results. Results indicate the number of positive droplets in each sample: 1, taken from infant’s Guthrie card on day 4 after birth, 0.49 parasites/µL; 2, taken from infant at hospital admission, 30,000 parasites/µL; 3, taken from infant at follow-up appointment, 599.8 parasites/µL; and 4, taken from mother at follow-up, 0.5 parasites/µL. FAM, fluorescein amidite.

We performed total DNA extraction using a QIAamp DNA Mini Kit (QIAGEN, http://www.qiagen.com) on dried blood spots on Whatman filter paper. We tested samples by endpoint nested PCR, as described by Singh (*8*). We performed molecular assays (Table 1) to assess parasite density, using standard curves prepared with 10-fold dilutions of DNA obtained from the *P. falciparum* 3D7 clone; result range was 10^4^ to 10^−1^ parasites/µL (Figure 1). We applied high-sensitivity quantitative PCR (qPCR) targeting the multicopy telomeric *var* genes to estimate the densities of the parasite. We performed digital PCR to confirm and compare results (Figure 2) (*5*).

**Table 1:** PCR amplification of *Plasmodium* spp. in study of malaria in mother and infant, Portugal*

Target	Primers	Assay type	Amplification conditions
18ssrRNA			
*Plasmodium* sp.	1st PCR reaction: rPLU forward primer, 5′-CTTGTTGTTGCCTTAAACTTC-3′; rPLU reverse primer, 5′-TTAAAATTGTTGCAGTTAAAACG-3′	Nested PCR	1× PCR master mix,† T1 Thermocycler–Biometic: 1 cycle, 95°C, 3 min); 30 cycles, 94°C, 1 min; 58°C, 1 min; 72°C, 1 min
*P*. *falciparum*	Nested PCR: Pf forward primer, 5′-TTAAACTGGTTTGGGAAAACCAAATATATT-3′; Pf reverse primer, 5′-ACACAATGAACTCAATCATGACTACCCGTC-3′ (Singh et al. [*8*])		1× PCR master mix,† 1 cycle, 95°C, 3 min; 35 cycles, 94°C, 1 min; 58°C, 1 min; 72°C, 1 min
*var*ATS	Forward primer, 5′-CCCATACACAACCAAYTGGA-3′; reverse primer, 5′-TTCGCACATATCTCTATGTCTATCT-3′; probe, 5′-6-FAM-TRTTCCATAAATGGT-NFQ-MGB-3′ (Hofmann et al. [*6*])	qPCR	1× TaqMan Gene Expression Mastermix,† 0.8 μM of each primer, 0.4 μM of probe; CFX96 Real-Time PCR Detection System‡: 1 cycle, 50°C, 2 min; 95°C 10 min; 45 cycles, 95°C, 15 sec; 55°C, 1 min
		dPCR	5x Absolute Q DNA Digital PCR Mastermix;§ Absolute Q Digital PCR§: 1 cycle, 50°C, 2 min; 95°C 10 min; 45 cycles, 95°C, 15 sec; 55°C, 1 min

*dPCR, digital PCR; qPCR, quantitative PCR. †Thermo Fisher Scientific, https://www.thermofisher.com. ‡Bio-Rad Laboratories, https://www.bio-rad.com. § Thermo Fisher, https://www.thermofisher.com

## Conclusions

This case highlights 2 key aspects for a better understanding of *P. falciparum* transmission: the role of imported, low-density asymptomatic cases as reservoirs and their potential contribution to congenital transmission. In Europe, ≈8,000 cases of imported malaria are reported annually (*2*); imported cases are mainly *P. falciparum* infections. Those populations can become a parasite reservoir that can pose significant risk to public health. Migrants may also display mild symptoms or be asymptomatic, often with submicroscopic parasitemia (*2*), which may be attributed to immunity acquired while residing in malaria-endemic regions (*9*). Such low levels of parasitemia can only be detected through sensitive molecular methods (*10*), such as qPCR-based techniques; we obtained the positive test result from this patient by nested PCR in the sample taken at hospital admission, when she was experiencing symptoms and parasitemia. In Portugal, there are potentially malaria-receptive areas and also a vector with some degree of competence (*3*). In non–malaria-endemic settings, availability of diagnostic tools varies by healthcare setting (Table 2). Nevertheless, clinicians should recognize the likelihood of congenital malaria and refer blood samples from suspected cases for testing.

**Table 2:** Diagnostic tools for *Plasmodium falciparum* detection in study of malaria in mother and infant, Portugal*

Diagnostic method	Limit of detection, parasites/μL	Level of care	Observations
Microscopy, thick smear	50–100	Secondary	Operator-dependent; limited sensitivity for low-density parasitemia
Rapid diagnostic test	100–200	Primary	Limited sensitivity for low-density parasitemia
Nested PCR, 18S rRNA target	1–5	Secondary/tertiary	Moderate sensitivity but may still miss low-density infections in neonates and mothers
qPCR, pfvarATS target	0.03	Tertiary	Multicopy gene enhances detection
dPCR pfvarATS target	≤0.01	Tertiary	Highest sensitivity, suitable for confirmatory diagnosis

*Sources: Dong et al. (*5*); Hofmann et al. (*6*); Singh et al. (*8*). dPCR, digital PCR; qPCR, quantitative PCR.

*var* genes are known for their role in antigenic variation, enabling *P. falciparum* to evade the host immune response. The high copy number of *var* genes can enhance the sensitivity of detection methods. For instance, the use of multicopy subtelomeric targets has been shown to improve the detection of low-density infections that might otherwise be missed using standard assays such as *18ssRNA* PCR (*6*). In our study, we established an accurate diagnosis for the mother through *pfvarATS* qPCR, which detected 0.03 parasites/µL, and digital PCR (dPCR), which detected 0.5 parasites/µL. Without the mother’s diagnosis, the child’s infection would have likely gone undetected and untreated. We have not established whether transmission to the infant was transplacental or from direct contact with maternal blood during labor. In most pregnancies resulting in congenital malaria, the mother’s infection tends to be symptomatic (*11*); malaria can also be diagnosed after uncomplicated asymptomatic pregnancies (*11*).

For our study, we defined congenital malaria as outlined in Oluput-Oluput (*12*) as the direct infection of an infant with malaria parasites from the mother before or during birth. Although all clinical indicators were consistent with congenital malaria, we pursued confirmation by detecting and quantifying parasite DNA in the Guthrie card sample taken on day 4 after birth. Both qPCR and dPCR confirmed the presence of *P. falciparum* parasites in the infant’s peripheral blood, with very low parasite densities (0.07 parasites/µL by qPCR and 0.49 parasites/µL by dPCR). Those findings align with the parasitemia levels observed in the mother, further supporting the likelihood of vertical transmission.

The delayed onset of symptoms in the infant can be attributed to several factors: the transfer of maternal antibodies through transplacental transfer and breastfeeding (*13*), low iron levels, and reduced erythropoiesis in newborns that do not favor *Plasmodium* spp. growth (*14*). Parasitemia might increase as maternal antibodies decline (*15*). In this case, the onset of symptoms occurred later than the previously reported median age, making the diagnosis more challenging (*7*). Furthermore, the nonspecific clinical signs of congenital malaria can be difficult to distinguish from other causes of sepsis (*4*). Our study highlights the importance of considering congenital malaria in the differential diagnosis of febrile infants born to mothers who have lived in malaria-endemic areas (*7*). We also emphasize the need for thorough analysis of blood smears in sepsis cases, particularly when thrombocytopenia is present (*12*).

In conclusion, this case underscores the utility of ultrasensitive detection targets and methods such as *pfvartATS* qPCR and dPCR for detecting submicroscopic malaria infections, particularly in asymptomatic migrant populations or populations at higher risk such as pregnant women and infants. Our findings emphasize the public health risk of overlooking hidden parasite reservoirs, which could hinder effective malaria control and prevention efforts in vulnerable groups.
